# Design of an Acoustic Bender Transducer for Active Sonobuoys

**DOI:** 10.3390/s19071691

**Published:** 2019-04-09

**Authors:** Seonghun Pyo, Hayeong Shim, Yongrae Roh

**Affiliations:** School of Mechanical Engineering, Kyungpook National University, Daegu 41566, Korea; nasgool@naver.com (S.P.); hiyo3@naver.com (H.S.)

**Keywords:** sonobuoy, bender transducer, acoustical characteristics

## Abstract

Recent underwater vehicles can operate with a much lower level of noise, which increases the need for an active sonobuoy with a high detection performance. These active sonobuoys mainly use bender transducers as a projector that emits sound waves. In this study, we designed a high-performance bender transducer and verified the validity of the design through experiments. For this purpose, first we analyzed the variation of the peak transmitting voltage response (TVR) level and peak TVR frequency of the bender transducer, in relation to its structural parameters. The performance of the bender transducer was analyzed using the finite element method. Then we derived the optimal structure of the bender transducer to achieve the highest TVR. Based on the design, a prototype of the bender transducer was fabricated and its acoustic properties were measured to confirm the validity of the design.

## 1. Introduction

Sonobuoys are expendable sonar devices that can be dropped from an aircraft or a ship into the ocean to perform underwater warfare sonar tasks [1,2,3,4,5,6]. The sonobuoy can be designed to perform as an active or a passive system [7,8,9,10]. Recent underwater vehicles can operate with a much lower level of noise, thus the need for an active sonobuoy with a high detection capability is increasing. Active sonar systems use one or more transmitters to produce an acoustic signal and multiple receivers to receive echoes of this acoustic signal from a target [11,12,13,14,15]. These sonobuoys generally use a modular projector system (MPS) if low-frequency underwater sound projection is desired. The MPS takes advantage of the interaction between closely spaced transducers to increase the radiation impedance and, thereby, increase power transmission [16]. The performance of a conventional underwater sound projector system is not very flexible. An MPS, on the other hand, offers flexibility in design because it allows a variety of inter-element spacing, as well as variation in individual transducer’s structure and size. The acoustic characteristics of the MPS, such as its resonance frequency and transmitting capability, can be controlled effectively through the design of individual transducers.

In general, various types of projectors, such as ring and barrel-stave type transducers, can be used as the elements of an MPS [16,17], but the most favorable choice is a flexural disk or a bender transducer [18]. The bender transducer is characterized by small size, a simple structure, and light weight. Several studies on the design and analysis of MPSs and bender transducers have been carried out over the past several years. Li et al. designed a line array of standard bender transducers to achieve a broadband MPS [19]. Wu and Wang reported a study on a multimorph transducer for application to the MPS [20]. Lei et al. analyzed the radiation interaction between bender transducers in an MPS [21]. The transmitting capability of an underwater projector is frequently restricted by the cavitation during high power transmission. Yamamoto et al. suggested differential connections of multiple disk bender transducers with different resonance frequencies to solve these problems [22]. However, all these investigations focused on the MPS system, not on individual bender transducers. Conventional bender transducers of a rather basic structure were used in these applications. If the structure of the bender transducer had been optimized for each application, the MPS might have achieved a better performance. Delany reported the empirical characterization of an existing bender transducer [23]. Recently, there was a paper on the design of a bender transducer [24], but there was a remarkable discrepancy between the designed and the measured transmitting performance of the transducer. The study to conduct a thorough analysis of bender transducer characteristics with verifiable validity to maximize the performance of the transducer has been very rare.

In this paper, we report on the design of the structure of a bender transducer through extensive structural analysis. We analyzed the transducer’s performance using the finite element method (FEM) in relation to various structural parameters and material properties. Then, we derived the optimal structure of the transducer to achieve the highest transmitting voltage response (TVR). A prototype of the bender transducer was fabricated based on this design and its characteristics were measured to verify the design’s validity [25].

## 2. Analysis of the Performance of the Bender Transducer in Relation to Structural Parameters

**Figure 1:**
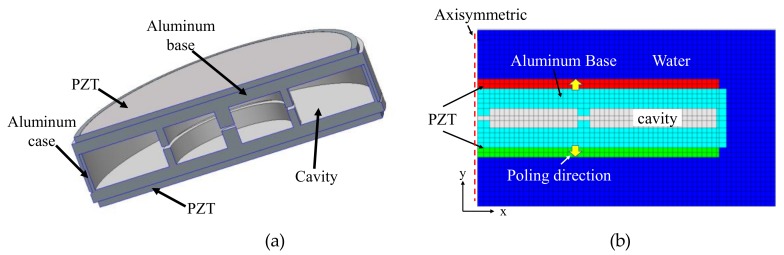
Bender transducer: (**a**) Schematic structure; (**b**) 2D axisymmetric FE model.

Figure 1a shows the basic structure of the bender transducer considered in this study. The transducer is comprised of two circular piezoelectric disks attached to the top and bottom surfaces of an aluminum case. The inner space of the aluminum case has two air cavities separated by two thin ribs, with the central rib being thicker than the other rib. The basic dimensions of the bender transducer are listed in Table 1. We used PZT5A for the piezoceramic material of the basic structure [26]. 

The acoustic characteristics of the bender transducer were investigated by means of the FEM using the commercial software package PZFlex^®^. Figure 1b is a 2D axisymmetric FE model of the transducer used in the analysis. A water domain with a radius of 700 mm was constructed around the bender transducer to model a radiation medium. The outer surfaces of the water were enforced with absorbing boundary conditions to prevent the reflection of the waves radiated by the transducer. The total number of elements, including those in water, was approximately 770,000. The dimension of each element was about 0.1% of the wavelength at the resonant frequency of the transducer, which was much smaller than the element size suggested by the PZFlex^®^. Such a fine mesh was implemented to achieve sufficient accuracy and stability of analysis results.

**Figure 2:**
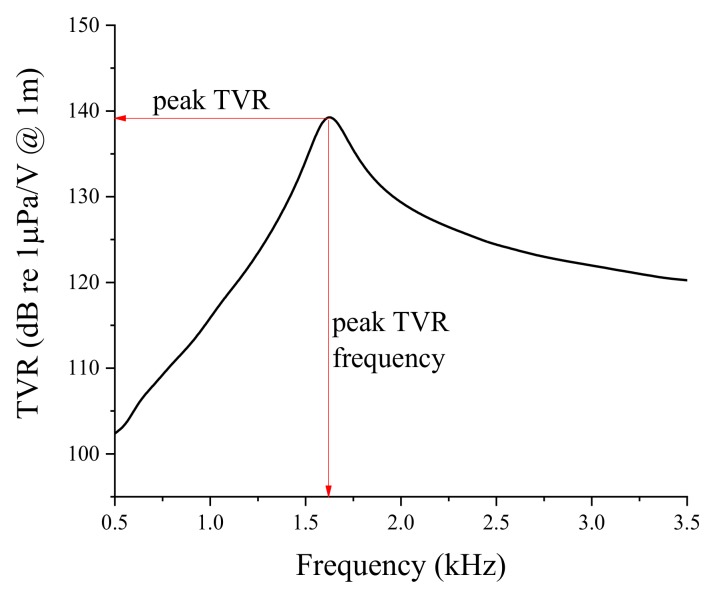
TVR spectrum of the bender transducer having the basic dimensions.

The transducer acoustic characteristics under consideration were the peak TVR level and the peak TVR frequency, because the transducer was to be used as an underwater projector. The underwater TVR was analyzed along the axis normal to the PZT disk. Figure 2 shows the TVR spectrum of a transducer having the basic dimensions in Table 1, which define the peak TVR level and the peak TVR frequency. The peak TVR frequency is the frequency at which the TVR has its peak value. In Figure 2, the peak TVR frequency of the basic bender transducer is 1.63 kHz and the peak TVR level is 139.2 dB. Variation of the TVR spectrum was analyzed by the FEM using the FE model in Figure 1b, in relation to various parameters of the transducer’s material and structure.

**Figure 3:**
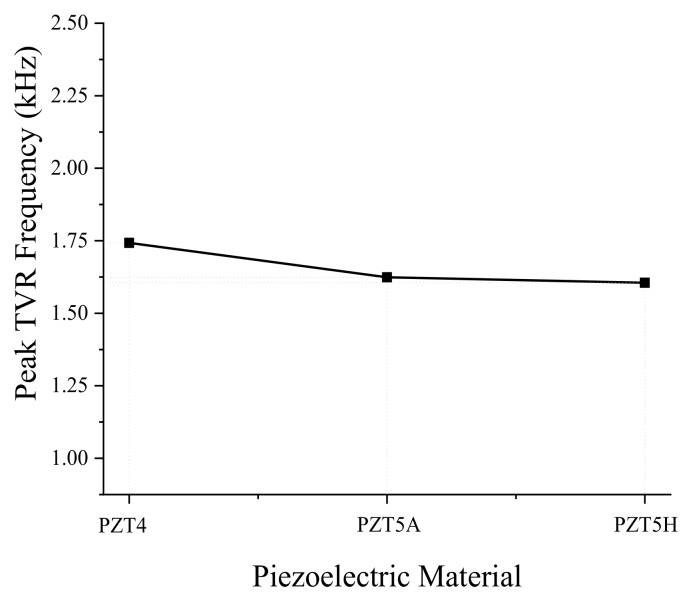
Effect of the piezoelectric material on the peak TVR frequency of the bender transducer.

**Figure 4:**
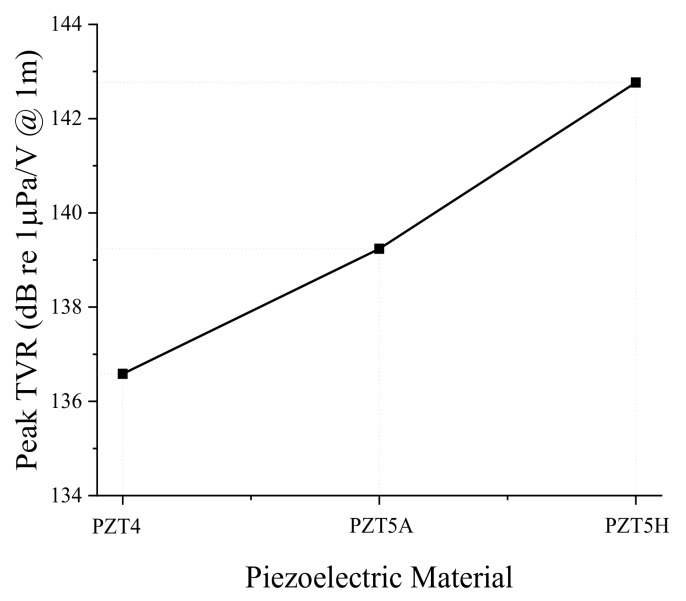
Effect of the piezoelectric material on the peak TVR level of the bender transducer.

First, we analyzed the effect of the piezoelectric material. When an electric voltage is applied to the piezoceramic plates, the plates vibrate in the radial direction, which causes the aluminum base placed between the piezoceramics to vibrate in the bending mode. As the most common piezoelectric materials available on the market, we tried PZT4, PZT5A, and PZT5H [26]. Results of the analysis are summarized in Figure 3 and Figure 4. The peak TVR frequency stayed almost constant for the different piezoelectric materials in Figure 3. However, the variation of the peak TVR level was significant, as shown in Figure 4. According to the constitutive equations of a piezoelectric material, the material property in charge of the radial mode vibration of the piezoceramic plate is the piezoelectric constant, d_31_ [27]. The d_31_ of PZT4, PZT5A, and PZT5H are −123, −171, and −274 pC/N, respectively. This mechanism explains why the peak TVR level is the highest for PZT5H, followed by PZT5A and then PZT4.

**Figure 5:**
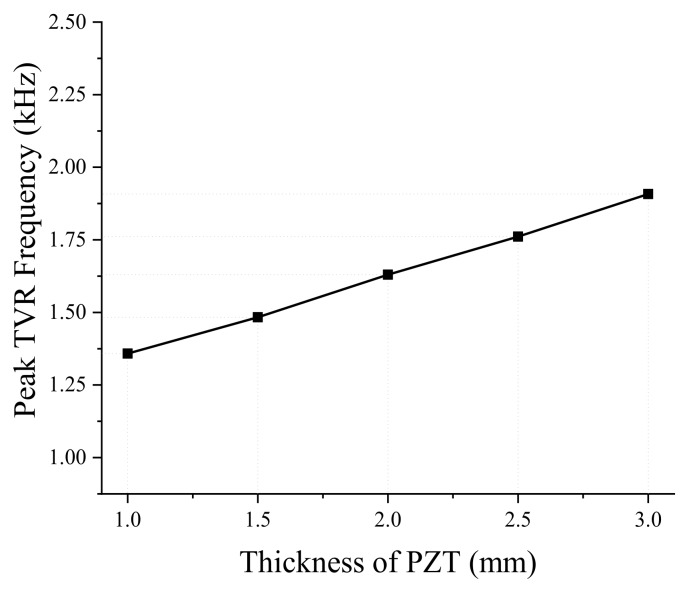
Effect of the PZT disk thickness on the peak TVR frequency.

**Figure 6:**
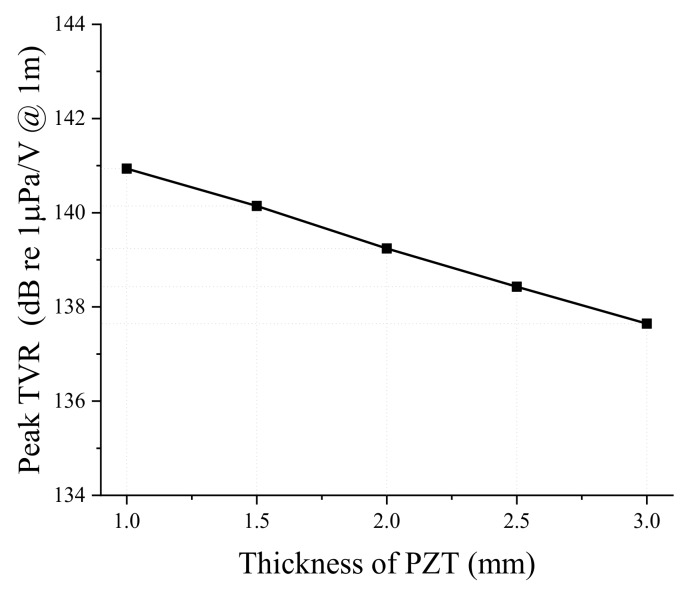
Effect of the PZT disk thickness on the peak TVR level.

**Figure 7:**
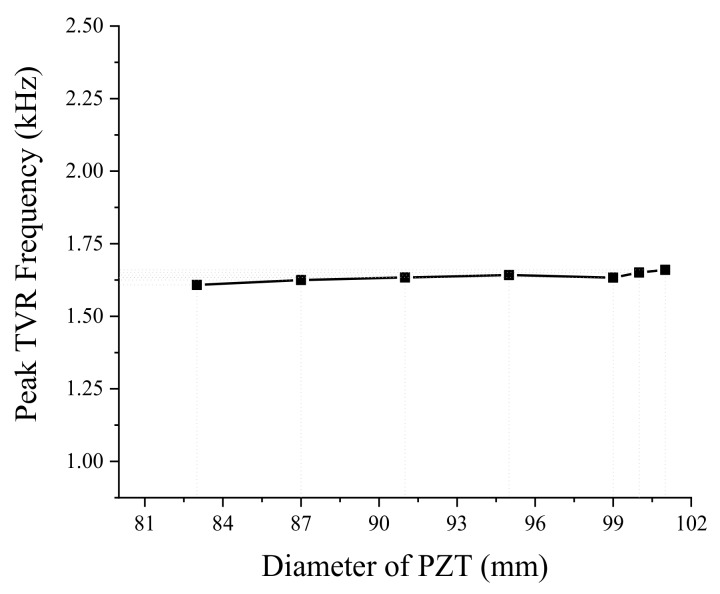
Effect of the PZT disk diameter on the peak TVR frequency.

**Figure 8:**
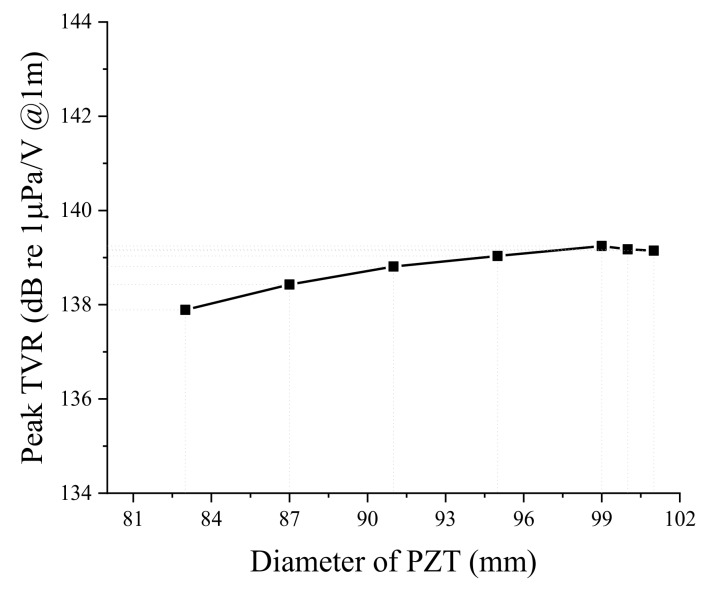
Effect of the PZT disk diameter on the peak TVR level.

**Figure 9:**
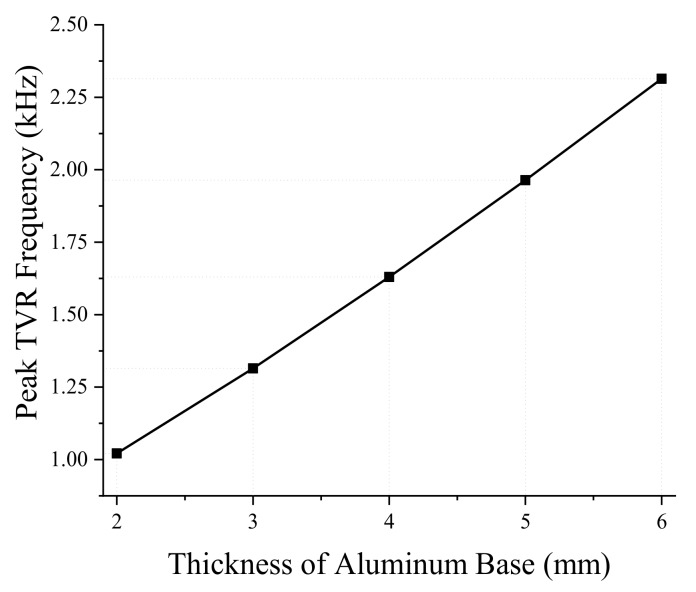
Effect of the aluminum base thickness on the peak TVR frequency.

**Figure 10:**
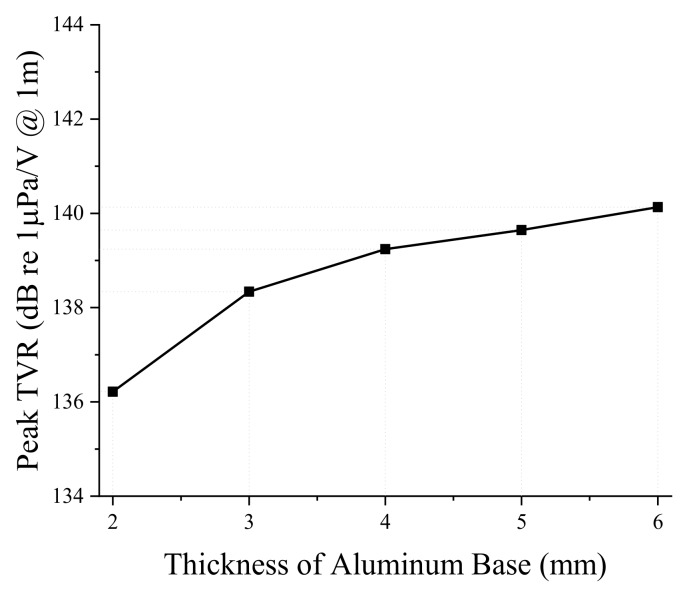
Effect of the aluminum base thickness on the peak TVR level.

Next, we analyzed the effect of dimensional variations on the performance of the bender transducer. Of the parameters listed in Table 1, the aluminum base inner and outer diameters were required to remain the same by the sonobuoy system requirements. We investigated the remaining eight structural parameters in Table 1 by the FEM, thickness and diameter of the PZT disk, thickness of the aluminum base, height of the rib and the cavity, inner and outer diameters of the small cavity, and the diameter of the center rib. According to the results, only the thickness and diameter of the PZT disk and thickness of the aluminum base turned out to have significant effects, as summarized in Figure 5, Figure 6, Figure 7, Figure 8, Figure 9 and Figure 10. The effects of the other parameters were negligible.

In Figure 5 and Figure 6, increasing PZT disk thickness leads to increasing peak TVR frequency but decreasing peak TVR level. An increase of the PZT disk thickness causes the whole transducer to be stiffer because PZT is stiffer than aluminum, thus the whole stiffness of the bender transducer increases, resulting in the higher peak TVR frequency. On the other hand, the deformation of piezoelectric material is proportional to the electric field along its thickness. For a constant electric voltage across the PZT disk, the larger thickness of the PZT disk means a smaller electric field over a unit thickness, which results in smaller deformation of the PZT disk. Hence, it is natural for the peak TVR level to decrease when the PZT disk thickness increases. 

In Figure 7, increasing the PZT disk diameter has no significant effect on the peak TVR frequency of the transducer. In Figure 8, the peak TVR level increases up to a certain diameter of the PZT disk and then decreases after that diameter. The main principle of operation of the bender transducer is the bending mode vibration of the aluminum base. The increase of the PZT disk diameter means more supply of electromechanical energy to the bender transducer for a constant input electric field, which results in a corresponding increase of the peak TVR level. However, a PZT disk with a diameter larger than a certain value seems to constrain the bending motion of the aluminum base, reducing the peak TVR level. 

In Figure 9 and Figure 10, increasing the aluminum base thickness leads to an increase in both the peak TVR frequency and the peak TVR levels. As the thickness of the aluminum base increases, the overall transducer becomes stiffer, which results in the higher peak TVR frequencies. The increase of aluminum base thickness also increases the peak TVR level by increasing the force emitted by the transducer to drive the bending mode of the aluminum bases.

## 3. Optimization of the Bender Transducer Structure

In the previous section, we verified that the three design variables, PZT disk thickness, aluminum base thickness, and PZT disk diameter, have a significant effect on the performance of the transducer. In this section, we keep the PZT disk thickness at its initial dimension. As the thickness of the PZT disk decreases, the TVR tends to increase, so a thinner PZT disk helps achieve a higher TVR. There is, however, another limitation to consider. The bender transducer we are designing here is to be used as an underwater projector and will be subject to a very high electric voltage. If the PZT disk becomes very thin, the voltage can damage it [28], so the disk will remain at its initial 2 mm thickness for this part of the design process.

In this section, we optimize the structure of the bender transducer to achieve the highest possible peak TVR level with the remaining two design variables, aluminum base thickness and PZT disk diameter. The variation range of each variable is shown in Table 2. The basic thickness of the aluminum base was set at 4 mm and the range of thickness variation was ±1 mm. Similarly, the basic diameter of the PZT disk was set at 99 mm and the range of diameter variation was ±2 mm. 

**Figure 11:**
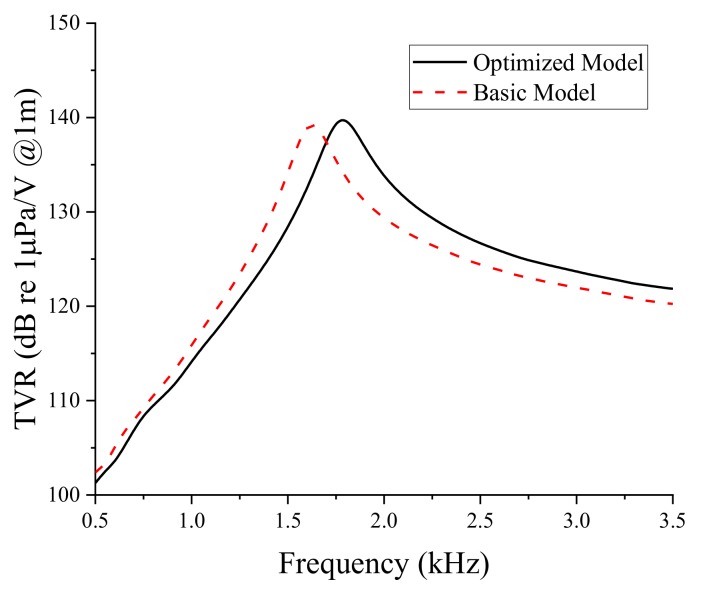
Comparison of the TVR spectra of the basic and optimized models of the bender transducer.

The objective function of the structural optimization was to maximize the TVR of the bender transducer. The constraint on the optimization was that the peak TVR frequency should be 1.8 kHz with a tolerance of ±20 Hz. Based on the results presented in Figure 7, Figure 8, Figure 9 and Figure 10, the optimum combination of the aluminum base thickness and PZT disk diameter was derived to maximize the TVR of the bender transducer using the OptQuest nonlinear programming (OQ-NLP) algorithm [29]. The OQ-NLP algorithm is a multistart heuristic algorithm to determine the global minimum of a constrained nonlinear problem. The optimized aluminum base thickness turned out to be 4.51 mm, with the PZT disk diameter at 98.9 mm. The TVR spectrum of the bender transducer having the optimized geometry is presented in Figure 11. The figure shows the TVR spectrum of the initial transducer as well, for comparison. Table 3 compares the transducer performance quantitatively. The peak TVR frequency of the optimized structure was 1.78 kHz, which satisfied the constraint on the peak TVR frequency. The peak TVR level of the optimized structure was 0.5 dB higher than that of the basic structure. The difference between the basic model and the optimized model was not large because the basic model was already elaborated through the analysis described in Section 2. 

## 4. Experimental Validation of the Design

**Figure 12:**
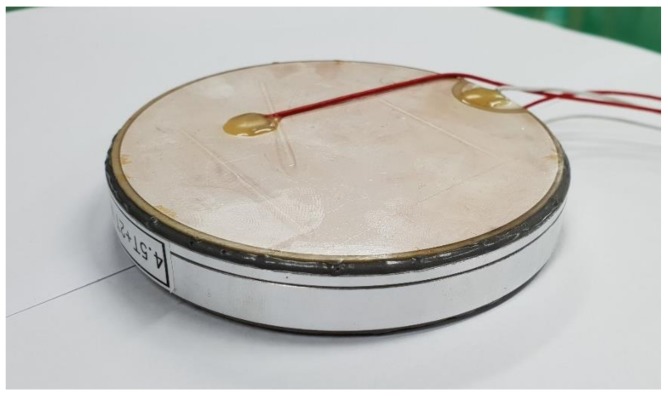
Photograph of the fabricated bender transducer.

A bender transducer having the optimal geometry was fabricated to validate its design. Figure 12 is a photograph of the unit. The dimensions of the transducer were the same as those in Table 1, except for the thickness of the aluminum base and the diameter of the PZT disk, which were the optimized values of 4.51 mm base thickness and 98.9 mm disk diameter. The manufacturing tolerance was controlled to be less than 0.1% for each structural parameter. The piezoelectric material was PZT5A. The impedance and the TVR spectra were measured and compared with those from the finite element analysis (FEA) of the transducer.

**Figure 13:**
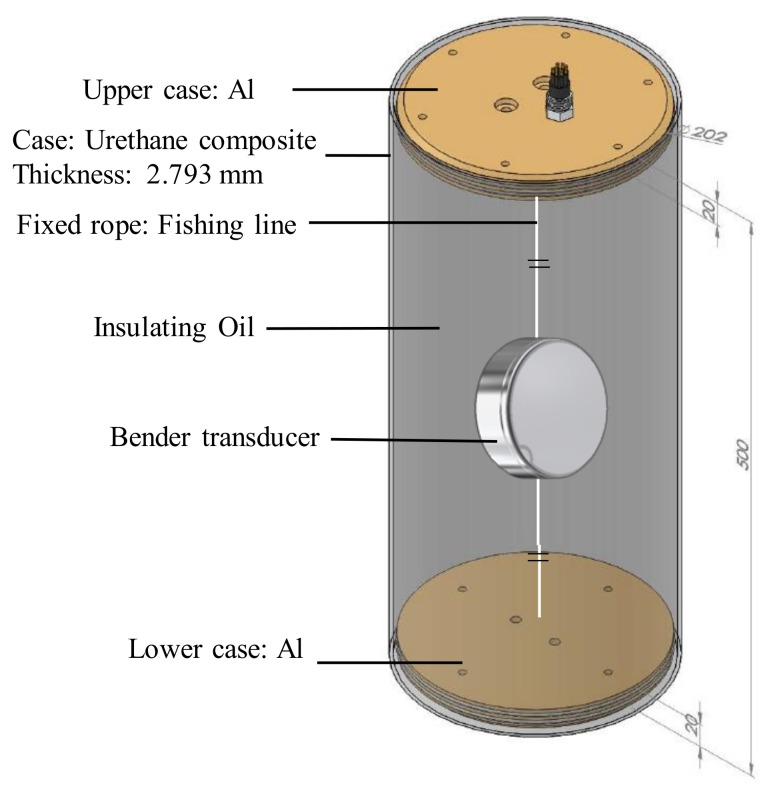
Schematic of the bender transducer characterization fixture.

**Figure 14:**
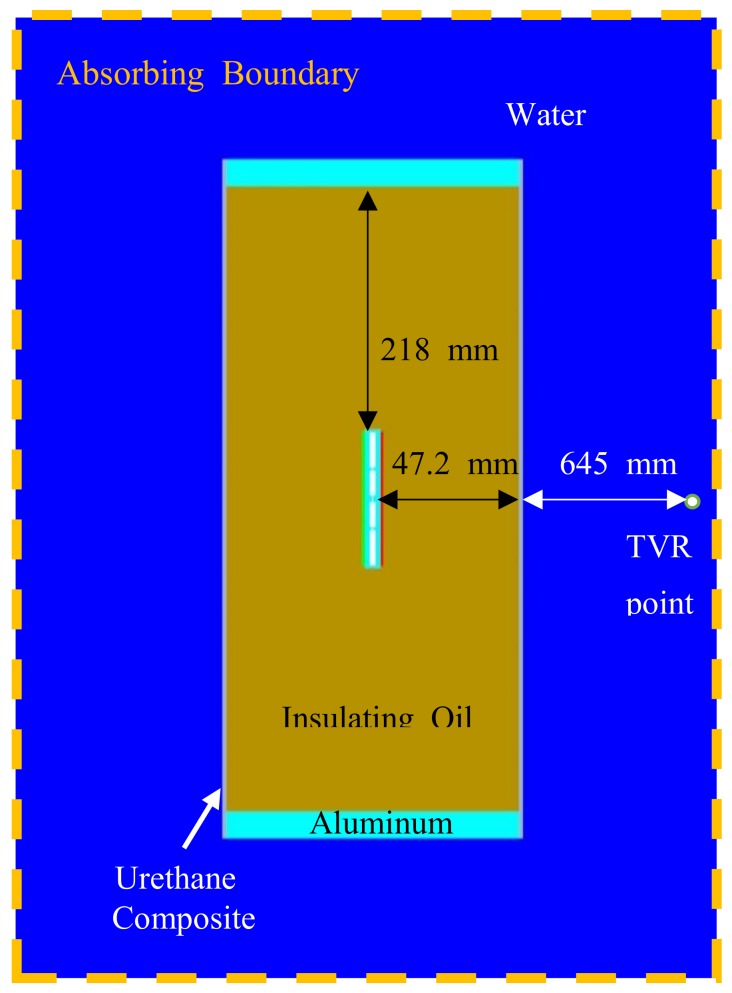
FE model of the bender transducer immersed inside the fixture.

In order to measure the underwater impedance and the TVR spectrum, we used the water-sealed fixture illustrated in Figure 13. The test fixture had an outer diameter of 202 mm, a length of 500 mm, and a thickness of 2.79 mm. A urethane composite was used as the covering material. The fixture with the bender transducer inside was filled with insulating oil. A circular aluminum base was mounted on the upper and lower surfaces to hold a thread to fix the bender transducer, as well as to mount the external hooks and electrical connectors. The diameter of the circular plate was 202 mm and the thickness was 20 mm. A FE model corresponding to the underwater testing fixture was developed to compare its results with the measured impedance and the TVR spectra of the bender transducer. Figure 14 shows the FE model for simulating the experimental measurement.

**Figure 15:**
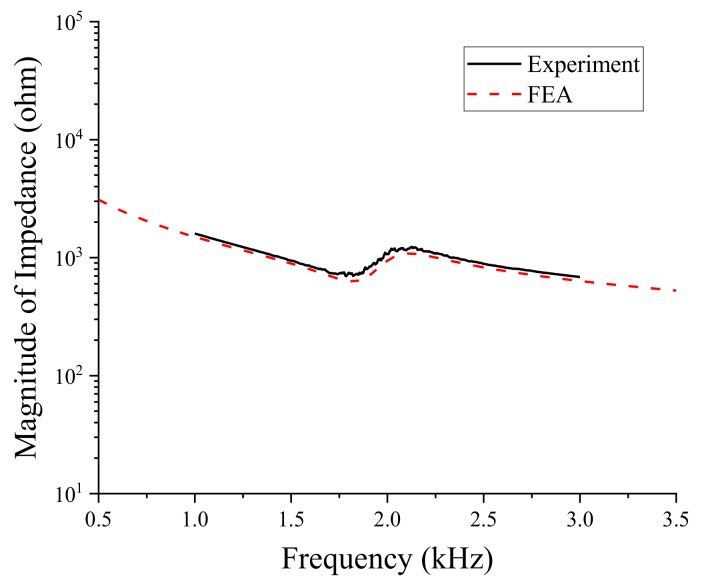
Comparison of the underwater impedance spectra from the FEA and measurement.

We measured the impedance spectrum of the bender transducer inside the test fixture with an impedance analyzer (Agilent 4294A). The result is compared with the impedance spectrum from FEA in Figure 15. The two spectra agree very well with each other. The quantitative difference in resonance and anti-resonance frequencies between the two spectra is less than 1%, as evidenced in Table 4. This validates the design in terms of the electromechanical properties of the bender transducer.

**Figure 16:**
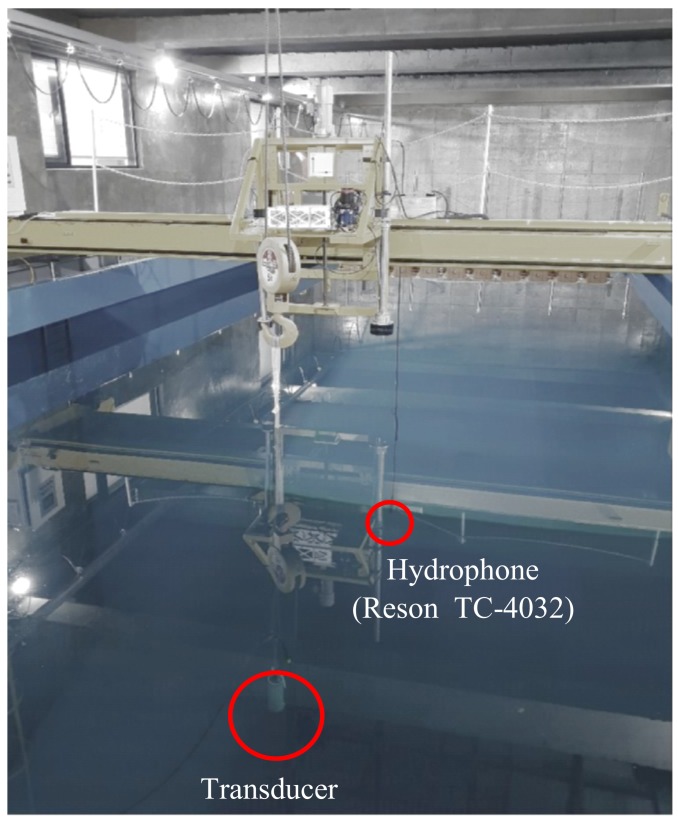
Photograph of the water tank measurement.

**Figure 17:**
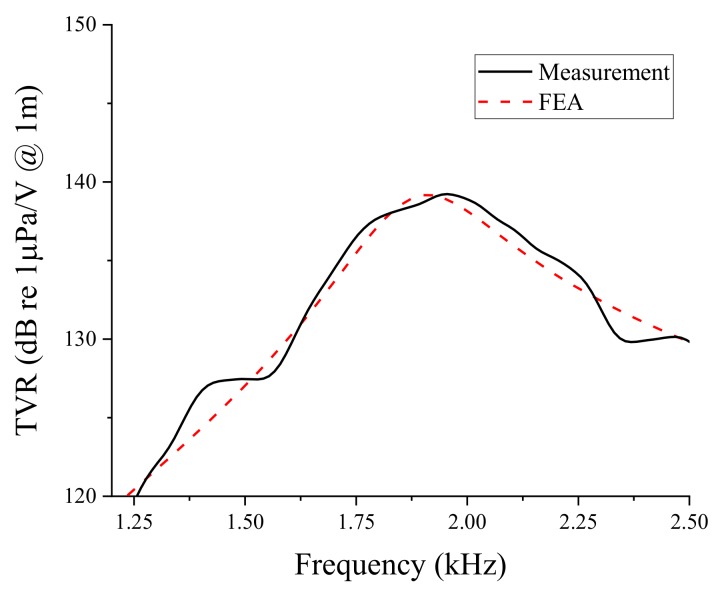
Comparison of the TVR spectra from the FEA and the measurement.

The TVR of the bender transducer mounted inside the test fixture was measured in a water tank, as illustrated in Figure 16. The bender transducer and hydrophone (Reson TC-4032) were installed 2.6 m apart at a depth of 4 m. The TVR spectrum from the measurement was compared with that from the FEA of the same transducer. The comparison in Figure 17 shows a good overall agreement between the two spectra. The quantitative performance from the measurement and the FEA of the transducer is listed in Table 5. The difference in the peak TVR frequency was merely 1.6%, while the peak TVR level was identical for both the measurement and FEA. This excellent agreement confirms the validity of the design of the bender transducer produced in this work. 

## 5. Conclusions

Recent underwater vehicles can operate with a much lower level of noise, which increases the need for an active sonobuoy with a high detection performance. However, most of the existing literature about active sonobuoys focuses on the MPS system, not on individual bender transducers. Hence, in this work, we conducted an extensive analysis of the performance of the bender transducer in relation to various structural parameters and material properties. Then, the optimal structure of the bender transducer was derived to achieve the highest TVR, while satisfying the constraint on the peak TVR frequency of the transducer. The validity of the design was verified by fabricating and characterizing a prototype bender transducer that had the optimized structure. Comparison of the performance from the measurement with the prototype transducer with that from the FEA of the transducer showed good agreement, which confirmed the validity and efficacy of the design. Considering that thorough analysis of the bender transducer characteristics with verified validity has been rare, the results in this work can be utilized to conduct a reliable design of various bender transducers for active sonobuoys to achieve more accurate underwater communication and detection.

## Figures and Tables

**Table 1:** Structural parameters for the bender transducer.

Parameter	Basic Dimension (mm)	Parameter	Basic Dimension (mm)
PZT disk thickness	2.0	Aluminum base thickness	4.0
PZT disk diameter	99.0	Aluminum base outer diameter	102.0
Total thickness of aluminum case	12.0	Aluminum base inner diameter	98.0
Center rib’s diameter	5.0	Small cavity outer diameter	46.0
Cavity height	4.0	Small cavity inner diameter	41.0

**Table 2:** Variation ranges of the design variables.

Design Variable	PZT Disk Diameter (mm)	Aluminum Base Thickness (mm)
Minimum dimension	97.0	3.0
Basic dimension	99.0	4.0
Maximum dimension	101.0	5.0

**Table 3:** Comparison of the acoustic characteristics of the basic and the optimized models of the bender transducer.

Characteristic	Basic Model	Optimized Model
Peak TVR frequency (kHz)	1.63	1.78
Peak TVR level (dB)	139.2	139.7

**Table 4:** Comparison of the resonance and anti-resonance frequencies from the FEA and the measurement.

Characteristic	FEA	Measurement
Resonance frequency (kHz)	1.82	1.82
Anti-resonance frequency (kHz)	2.11	2.13

**Table 5:** Comparison of the peak TVR frequency and peak TVR level from the FEA and the measurement.

Characteristic	FEA	Measurement
Peak TVR frequency (kHz)	1.92	1.95
Peak TVR level (dB)	139.2	139.2
